# Boost Approaches in Patients Undergoing Postoperative Radiotherapy

**DOI:** 10.1002/jso.70228

**Published:** 2026-03-08

**Authors:** Abigail Pepin, Neil D. Almeida, Simon Fung‐Kee‐Fung, Megan Kassick, Neil K. Taunk, Gary M. Freedman

**Affiliations:** ^1^ Department of Radiation Oncology Perelman School of Medicine Philadelphia Pennsylvania USA; ^2^ Department of Radiation Oncology Roswell Park Comprehensive Cancer Center Buffalo New York USA

**Keywords:** breast boost, breast cancer, concurrent boost, radiation, sequential boost

## Abstract

The use of breast conservation surgery for early‐stage breast cancer is common, and adjuvant radiotherapy is often recommended to reduce the risk of ipsilateral breast tumor recurrence. Historical trials have used sequential boost techniques with favorable outcomes with reduction in local recurrence and acceptable cosmetic outcomes. In recent years, there has been renewed interest in incorporating a concurrent tumor bed boost to reduce the number of radiation treatments delivered. This focused review summarizes the literature surrounding the indications, outcomes, and treatment planning considerations for radiation tumor bed boost for early‐stage breast cancer patients.

## 1 Introduction

Breast cancer is the most frequently diagnosed non‐skin cancer in women [1]. Over time, there has been increasing utilization of breast conservation surgery (BCS) for patients with early‐stage breast cancer [2, 3]. Adjuvant radiotherapy is recommended for the overwhelming majority of patients who undergo BCS. The early data from multiple large randomized control trials of post‐lumpectomy whole breast radiotherapy (WBRT) demonstrated significantly improved local control and a 15‐year absolute risk reduction in all‐cause mortality of 3% compared to no adjuvant radiotherapy [4]. Despite these advances, uncertainty remained as to whether dose escalation in addition to WBRT, also called a lumpectomy bed boost, is routinely needed for patients with negative margins following BCS. Notably, the landmark B‐06 trial, which demonstrated reduction in risk of ipsilateral breast tumor recurrence (IBTR) with the use of adjuvant radiation, did not use a tumor bed boost (TBB) [5]. The rationale of using a boost is that local recurrence occurs most commonly at the margins of the lumpectomy bed likely due to the higher chance of microscopic tumor cells at these locations [6]. A radiation TBB to the area at high risk of microscopic residual disease may more effectively eradicate microscopic disease and thus lower the risk of IBTR [6]. Several randomized trials investigating the use of TBB demonstrated a significant decrease in IBTR [7, 8, 9, 10]. In one meta‐analysis, the employment of TBB was found to have an improvement in IBTR (HR 0.64), though it did not directly impact disease‐free survival (DFS) or overall survival (OS) [6].

In this review, we discuss the current status of the post‐BCS TBB for early‐stage breast cancer. We examine the efficacy of TBB on IBTR, indications for TBB, cosmetic outcomes of TBB with standard and hypofractionated radiation, and outcomes using simultaneous integrated boost (SIB) approach. Lastly, we discuss radiation technique and technical considerations in planning and delivering the radiation boost.

## 2 Efficacy of a Tumor Bed Boost

In the mid‐1980s to 1990s, several prospective randomized control trials investigated the role of TBB after whole breast radiation (summarized in Table 1). The landmark EORTC 22881‐10882 trial heralded in a new era of routine use of the breast boost in the setting of BCS. Patients were randomized following conventional WBRT to 50 Gy in 25 fractions to either 16 Gy photon TBB (or 15 Gy with an iridium‐192 implant) or no boost [11]. With a median follow‐up of 17.2 years, there was a significant reduction in IBTR (20‐year cumulative incidence 16.4% no boost arm versus 12% in the boost arm, *p *< 0.0001). There was no difference in OS (*p *= 0.323) [11]. Only younger age groups (< 50 years) had a significant relative reduction in IBTR with a boost dose [11]. A separate analysis did not demonstrate any differences in 5‐year IBTR between the electron, photon, and interstitial boost arms [12].

**Table 1:** Efficacy of tumor bed boost in the setting of boost versus no boost and dose escalation.

Trial	*N*	Years of enrollment	Median follow‐up	Inclusion criteria	Randomization	Findings
EORTC 22881‐10882 [11]	2657	1989–1996	17.2 years	T1‐2 N0‐1 1–2 cm margin required	50 Gy in 25 fractions ± sequential 16 Gy boost	20‐year cumulative IBTR: 16.4% (no boost) vs. 12% (boost arm), *p* < 0.0001 20‐year OS: 59.7% (boost) vs. 61.1% (no boost), *p* = 0.323 20‐year severe fibrosis rate: 1.8% (no boost) vs. 5.2%, *p* < 0.0001
Lyon [7]	1024	1986–1992	3.3 years	Invasive ductal carcinoma ≤ 3 cm Negative margins (no tumor on ink) < 70 years	50 Gy in 20 fractions WBRT ± sequential 10 Gy in 4 fractions boost (with 9 or 12 MeV)	5‐year local relapse: 3.6% (boost) vs. 4.5% (no boost), *p* = 0.044 Grades 1–2 telangiectasias: 5.9% (no boost) vs. 12.4% (boost)
Budapest [8]	207	1995–1998	5.3 years	T1‐2, N0‐1	50 Gy in 25 fractions WBRT ± sequential 16 Gy electron boost or 12–14.25 Gy HDR BT boost	5‐year IBTR: 92.7% (boost) vs. 84.9% (no boost), *p* = 0.049 5‐year RFS: 76.6% (boost) vs. 66.2% (no boost), *p* = 0.044 5‐year CSS: 90.4% (boost) vs. 82.1% (no boost), *p* = 0.053 No difference between good to excellent cosmetic results between boost vs. no boost No difference between cosmetic outcomes between HDR boost vs electron boost
Nice [9]	664	1987–1994	73 months	Invasive ductal cancer Negative margins	50 Gy in 25 fractions ± sequential 10 Gy in 5 fractions	Local recurrence: 6.8% (no boost) vs. 4.3% (boost), *p* = 0.13 Median delay of local recurrence: 26 (no boost) vs. 50 months (boost), *p* = 0.05
Australian St. George and Wollongong Trial [10]	688		8.5 years	Tis‐2 N0‐3	50 Gy in 25 fractions vs. 45 Gy in 25 fractions + sequential 16 Gy in 8 fraction boost	5‐year IBTR rates were 2.5% (no boost) vs. 3% (boost) Reduced whole breast dose was associated with inferior IBTR despite the use of a boost
BIG 3‐07/TROG 07.01	1608	2007–2014	6.6 years	DCIS At least 1 mm of clear radial resection margins At least one: ◦ < 50 years ◦ Multifocal disease ◦ Intermediate or high nuclear grade ◦ Central necrosis ◦ Radial surgical margin < 10 mm	4 arms: 50 Gy in 25 vs. 42.5 Gy in 16 with or without 16 Gy in 8 fractions	5‐year freedom from local recurrence: 92.7% (no boost) vs. 97.1% (boost), *p* < 0.001 Grade 2+ breast pain: 10% (no boost) vs. 14% (boost), *p* = 0.003 Induration 6% (no boost) vs. 14% (boost), *p* < 0.001
“Young Boost”	2421	2004–2011	11.7 years	≤ 50 years pT1‐2 N02‐a ECOG ≤ 2 Microscopically complete or focally involved resection	50 Gy in 25 fractions + 16 Gy vs. 26 Gy boost	10‐year local recurrence: 4.4% (low dose boost) vs. 2.8% (high dose boost), *p* = 0.032* *Primary end point that high radiation boost after whole breast irradiation improves local control by 3.5% not met
IMPORT HIGH	2617	2009–2015	74 months	pT1‐3 N0‐3a Clear microscopic resection	40 Gy in 15 fractions with: 1. Sequential 16 Gy in 8 fraction boost 2. 48 Gy in 15 fraction boost 3. 53 Gy in 15 fraction boost	5‐year IBTR: 1.9% (sequential boost), 2.0% (48 Gy concurrent boost), 3.2% (53 Gy concurrent boost) 5‐year incidence of moderate or marked breast induration: 11.5%, 10.6% (*p *= 0.4 vs. control), 15.5% (*p *= 0.015 vs. control)

The Lyon group also conducted a randomized trial of early‐stage breast cancer patients treated with BCS followed by adjuvant WBRT with or without a boost (Table 1). At 5 years, they demonstrated reduced local relapse rates using the boost (3.6% boost, 4.5% no boost) and time to local recurrence favored the boost group (*p *= 0.044) [7]. The Budapest group randomized women with early‐stage breast cancer who underwent BCS and WBRT to receive no further radiation or an additional electron or high dose rate (HDR) brachytherapy TBB (Table 1) [8]. The 5‐year results demonstrated an improvement in IBTR (92.7% vs. 84.9%, *p* = 0.049), relapse‐free survival (76.6% vs. 66.2%, *p* = 0.044), and cancer‐specific survival (90.4% vs. 82.1%, *p* = 0.053), favoring the boost arm [8]. In subgroup analysis, the actuarial 5‐year local recurrence rate for patients under 40 years was 27.1% versus 34.4% in the boost versus no boost arms, respectively (*p* = NS) [8]. The Nice trial, which is available only in abstract form, randomized patients treated with adjuvant WBRT to no boost versus an additional 10 Gy in 5 fraction boost. At a median follow‐up of 6 years, there was no difference in IBTR between the arms (6.8 vs. 4.3%, Table 1) [9]. Lastly, the Australian St. George and Wollongong trial randomized patients with early‐stage breast cancer to 50 Gy in 25 fractions to the whole breast versus 45 Gy in 25 fractions to the whole breast followed by a 16 Gy in 8 fraction electron boost (Table 1). At a median follow‐up of 8.5 years, the 5‐year IBTR rates were 2.5% versus 3% for the no boost versus boost arms. The authors concluded that reducing the whole breast dose was associated with inferior IBTR, despite the use of a boost [10]. A meta‐analysis performed of these five Phase III trials analyzing 8235 women treated in the 1980s–1990s demonstrated that local control was improved with the administration of a TBB (HR 0.64) [6]. This local control benefit did not translate into an OS or DFS benefit [6].

Sequential boost has also been examined in the setting of ductal carcinoma in situ (DCIS). The BIG 3‐07/TROG 07.01 trial was a multicenter, Phase III study, which randomized patients undergoing WBRT (either 50 Gy in 25 fractions or 42.5 Gy in 16 fractions) to receive a sequential boost (16 Gy in 8 fractions) or no boost [13]. The boost arm had a higher 5‐year freedom‐from‐local recurrence (92.7% no boost, 97.1% boost) for patients with at least one high risk feature including age < 50 years old, Grades 2–3 disease, presence of necrosis, or margins < 1 cm (Table 1).

More modern trials have the efficacy of boost dose escalation (Table 1) beyond the standard 10–16 Gy in 2 Gy fractions. These trials have not demonstrated a benefit for dose escalation. For example, the “Young Boost trial” investigated the impact of high dose (26 Gy) versus standard dose (16 Gy) sequential boost with respect to local recurrence [14]. This trial did not demonstrate a significant reduction in IBTR with high radiation boost (4.4% high dose vs. 2.8% low dose). The IMPORT HIGH trial randomized women to 40 Gy in 15 fractions with either a sequential boost (16 Gy in 8 fractions) or a concurrent boost (48 Gy in 15 fractions or 53 Gy in 15 fractions). Similar to the Young Boost Trial, there was no reduction in 5‐year IBTR rates between the arms (1.9% sequential boost, 2% 48 Gy concurrent boost, 3.2% 53 Gy concurrent boost) [15]. The EORTC 22881‐10882 trial evaluated dose escalated TBB in the setting of microscopically positive margins. Patients with positive margins were randomized to a standard dose boost (10 Gy) versus a high dose boost (26 Gy). At 10 years, the cumulative local recurrence rate was 17.5% for standard dose boost versus 10.8% high dose boost, which was not statistically significant (*p *> 0.1) [16]. These trials demonstrated that dose escalation did not improve IBTR and resulted in worsened cosmetic outcomes.

## 3 Indications for Boost

Analysis of several clinical trials demonstrated that younger age, high tumor grade, extensive intraductal component, receptor status, and positive surgical margins are risk factors for IBTR. The EORTC 22881‐10882 boost trial demonstrated an improvement in local control amongst all patients receiving TBB (HR 0.55) [17]. Younger patients have a higher risk of local recurrence, and thus, the absolute reduction of local recurrences with TBB is highest amongst patients younger than 50 years of age. For women < 40 years old, the 20‐year risk of IBTR was 36% without a TBB and 24.4% with a TBB (absolute reduction 12% in IBTR with TBB) [11]. Similarly, amongst patients aged 41–50 years old, IBTR was 13.2% without a boost and 9.7% with a boost (absolute reduction of 3.5%) [11]. Subsequent analyses of the EORTC boost trial demonstrated that high‐grade tumors and those associated with DCIS had higher rates of IBTR [18, 19]. Receptor status and molecular subtyping play additional roles in IBTR. A meta‐analysis of 15 studies in 12 592 breast cancer patients treated from 1980 to 2008 demonstrated that luminal subtype tumors (ER/PR positive) were less likely to develop a local recurrence following BCS relative to patients with triple negative (RR 0.61) or HER2/neu overexpressing tumors (RR 0.34). Similarly, higher Oncotype DX scores are independently associated with increased risk of locoregional recurrence even when accounting for other risk factors [20]. Further, surgical margin is a known risk factor for IBTR. For example, in the Budapest trial, young age (< 40 vs. ≥ 40), positive margin status, and high mitotic activity index had a significant negative effect on local tumor control [8]. Separate analysis of patients with positive or close margins demonstrated an improvement in 5‐year actuarial breast relapse rate from 50.8% to 8.3% with boost (*p *= 0.04) [8]. A meta‐analysis of 33 studies of patients treated from 1979 to 2001 suggested the odds of local recurrence were significantly higher with positive margins (OR 1.96 positive close vs. negative) [21]. This is consistent with other reported analyses in the literature, which suggest a two‐fold increased risk of IBTR compared to negative margins [22]. Although boost may altogether reduce the risk of IBTR, the risk of IBTR with positive margins remained elevated despite the use of boost or dose‐escalated boost [21]. Further, there is no evidence that widely clear margins reduces the risk of IBTR; hence, the use of “no tumor on ink” is the standard for an adequate margin in the setting of invasive cancer [21, 22].

With modern imaging and surgical techniques, IBTR rates have substantially declined from the boost versus no‐boost era. Hence, TBB may not be necessary for all patients and may offer different cosmetic outcomes (CO). In patients without risk factors for local recurrence, the 10‐year rates of IBTR on modern trials are low without TBB ranging from 1.3% to 2.8% [23, 24]. The DBGG PBI trial, which employed 40 Gy in 15 fractions to the whole breast or partial breast, also reported low rates of 9‐year locoregional recurrences (1.7% WBRT, 3.1% partial breast) [25]. These trials enrolled women at low risk of breast cancer recurrence (e.g., pT1‐2, pN0, > 50–60 years, luminal subtype). In the START B trial, nearly 60% of patients did not receive a TBB [26]. In the EORTC boost trial, a boost was omitted in patients over the age of 50 with low‐grade tumors [11]. As previously mentioned, there is limited data to suggest that even close margins benefit from a TBB.

The NCCN guidelines recommend a TBB in patients at elevated risk of IBTR including those patients younger than 50, with high‐grade disease, or with focally positive margins [27]. The ASTRO consensus statement recommends a TBB for patients with higher risk features including those younger than 50 years of age with any grade disease, those aged 51–70 with high‐grade disease, or those who have a positive resection margin [28]. In patients with DCIS, a TBB may be omitted if a patient is > 50 years or has a low‐grade disease or negative margins. Current guidelines recommend omission of a TBB in patients deemed low risk (i.e., > 70 years with low or intermediate grade and widely negative margins). Per the ASTRO task force, evidence is strongest in favor of sequential boost after WBRT and is the current recommendation outside of clinical trials. Notably, this recommendation was published in 2018 and includes literature through May 2016. Hence, the recommendation predates major clinical trials using a SIB approach.

We acknowledge there are areas of uncertainty surrounding TBB and the associated benefit to patients. This includes patients who receive neoadjuvant chemotherapy and achieve a pCR as those individuals may have low rates of IBTR at baseline and the magnitude of benefit from adjuvant radiation and/or a TBB may be limited. This is an area of ongoing investigation (e.g., NRG BR008 HERO trial) [29]. Ultimately, the decision on utilization of TBB relies on shared decision‐making between the provider and the patient, based on patient factors, tumor characteristics, treatment factors, and patient preferences.

## 4 Cosmetic Effects of a Boost

There are potential concerns regarding the long‐term CO of a TBB. This may influence individual patient decision‐making for a boost when weighing the potential risks against the benefits. The cosmetic outcomes among patients enrolled in the trials examining boost versus no boost are highlighted in Table 1.

The EORTC 22881 trial demonstrated a higher incidence of severe fibrosis at 20 years in the boost arm relative to the nonboosted arm (1.8% vs. 5.2%, *p* < 0.0001) [11]. The high‐dose boost versus low‐dose boost subgroup analysis revealed higher rates of severe palpable fibrosis in the high dose and boost areas [16]. Determinants of poorer CO include boost treatment itself, larger excision volumes, younger age, tumors in the central lower quadrants of the breast, and a boost dose administered with photons [30, 31]. Another analysis of the EORTC boost trial failed to demonstrate any difference in 5‐year fibrosis rates between different boost techniques (i.e., electrons, photons, interstitial) [12]. The Budapest group demonstrated similar rates of good to excellent cosmetic results irrespective of no boost versus boost (*p *= 0.14) and technique (HDR brachytherapy boost versus electron boost, *p* = 0.29) [8]. The Lyon group demonstrated an improvement in local control was at the cost of increased Grades 1–2 skin telangiectasias (12.4% vs. 5.9%) [7]. The St. George and Wollongong trial examined overall CO by panel and BCCT.core software and found numerically worse pBRA (relative breast retraction assessment) and absolute breast retraction assessment scores, though these were not statistically significant [32]. They also found patient‐reported outcomes were excellent and good in 95% and 93% of the boost and no‐boost arms, respectively [32]. Analysis of cosmetic status in DCIS patients on the BIG 3‐07/TROG 07.01 trial demonstrated worse cosmetic outcomes with TBB relative to no boost across all time points, which persisted until at least 24 months [33]. A meta‐analysis looking at panel‐scored cosmesis demonstrated superior cosmesis outcomes in patients without a TBB (*p *= 0.01), but physician‐scored cosmesis did not differ between the boost and no‐boost groups (*p *= 0.09).

Further, boost dose escalation may increase risk of inferior cosmetic outcomes. Results of the Young Boost trial revealed CO was significantly better in the standard boost group at 4 years compared to the dose escalated boost cohort [14]. Similarly, dose escalation in the IMPORT HIGH trial demonstrated inferior cumulative incidence of clinician‐reported moderate to marked breast induration at 5 years with dose escalated hypofractionated SIB (11.5% no boost, 10.6% 48 Gy in 15 fractions concurrent boost, 15.5% 53 Gy in 15 fractions concurrent boost) [15].

Given the worse cosmesis in cases of boost over nonboost and dose escalation, there was interest in examining cosmetic effects in the setting of hypofractionation. While the trials to date report overall cosmesis in the setting of hypofractionation, they compare this cosmesis to other standard boost options rather than no boost. Current evidence does not suggest increased detrimental cosmetic effects in the setting of hypofractionation. For example, the DBCG HYPO Trial randomized patients to receive WBRT to 50 Gy in 25 fractions or 40 Gy in 15 fractions followed by a sequential boost of 10–16 Gy in 2 Gy fractions per the discretion of the enrolling institution [34]. Patients treated with boost had less induration at baseline, but the boost did not increase the risk of induration with longer follow‐up. For patients treated with and without a boost, the 3‐year incidence of induration was 12% (50 Gy, boost) and 8% (40 Gy, boost) and 13% (50 Gy, no boost) and 11% (40 Gy, no boost), respectively. In the multicenter French HypoG‐01 trial, patients received 40 Gy in 15 fraction versus a 50 Gy in 25 fraction ± sequential or concurrent boost per the investigator's discretion [35]. In their cohort, most patients received a sequential boost (67.8%). Grade ≥ 2 dermatitis was more frequent in patients who received a TBB, though Grade ≥ 2 dermatitis was numerically less frequent among those who received the hypofractionated course (20.5% vs. 9.1% in 3‐week arm, 46.4% vs*.* 17% in 5‐week arm) [35]. Similarly, patients who underwent concurrent boost had numerically lower rates of Grade ≥ 2 dermatitis compared to those who underwent a sequential boost (concurrent: 13.1% 3‐week, 29.2% 5‐week; sequential: 24.2% 3‐week, 54.2% 5‐week) [35].

There are more limited data on cosmetic outcomes of TBB in the setting of ultra‐hypofractionation. In the FAST FORWARD trial, approximately 25% of patients received a TBB. While the study does not report on cosmetic differences between the boost patients, the ultra‐hypofractionated trial population demonstrated acceptable cosmetic outcomes [36]. Single institutional data from Switzerland examining patients treated with ultra‐hypofractionated 26 Gy in 5 fractions with or without a sequential boost of 10–12.5 Gy has been reported. In that series, 97.7% of patients reported good or excellent cosmetic results. There were no significant differences in fair cosmetic outcomes observed among patients treated with or without boost (RR = 1.016) [37]. Another series out of Memorial Sloan Kettering compared patients treated with 26 Gy in 5 fractions with or without a single fraction cavity boost. Approximately 59% of patients received a boost, and the receipt of a boost was not associated with a higher risk of toxicity in their series [38].

## 5 Concurrent Boost

The widespread adoption of hypofractionated WBRT has significantly reduced the time spent on radiation treatment for most breast cancer patients. Further reduction of treatment days may be achieved by delivering a TBB concurrently with WBRT (SIB). There have been a large number of Phase II trials that have demonstrated safety and efficacy and four Phase III prospective randomized trials [39]. These trials are summarized in Table 2.

**Table 2:** Summary of simultaneous integrated boost (SIB) trials.

Trial	*N*	Years of enrollment	Median follow‐up	Inclusion criteria	Randomization	Findings
IMRT‐MC2 [40]	502	2011–2015	5.1	Age 18–70 OR > 70 with at least one risk factor: ◦ ≥ T2 ◦ Multifocal disease ◦ LVSI ◦ EIC ◦ Margins < 3mm	Randomized to either: IMRT: 50.4 Gy in 28 fractions with SIB to 64.4 Gy 3D‐CRT: 50.8 Gy in 28 fractions with sequential dose to 66.4 Gy	2‐year local control: 99.6% vs. 99.6%, *p *= 0.487 Noninferiority for cosmesis after IMRT‐SIB and 3D‐CRT‐sequential boost at 6 weeks and 2 years
RTOG 1005 [34]	2262	2011–2014	7.3 years	DCIS, Grade 3, and < 50 years OR ypStage 0, I, II breast cancer after BCS after neoadjuvant chemotherapy	50 Gy in 25 fractions or 42.7 Gy in 16 fractions with sequential boost of 12 Gy in 6 versus 40 Gy in 15 fractions with concomitant boost to 48 Gy	7‐year IBRT: 1.9% (sequential boost) vs. 2.6% (concurrent boost) No difference in 3‐year excellent/good cosmesis: 86% (sequential boost) vs. 84% (concurrent boost) No significant differences in grade 3+ toxicity (*p *= 0.79)
IMPORT HIGH [41]	2617	2009–2015	74 months	pT1‐3 N0‐3a Clear microscopic resection	40 Gy in 15 fractions with: 1. Sequential 16 Gy in 8 fraction boost 2. 48 Gy in 15 fraction boost 3. 53 Gy in 15 fraction boost	5‐year IBTR: 1.9% (sequential boost), 2.0% (48 Gy concurrent boost), 3.2% (53 Gy concurrent boost) 5‐year incidence of moderate or marked breast induration: 11.5%, 10.6% (*p *= 0.4 vs. control), 15.5% (*p *= 0.015 vs. control)
HYPOSIB [35]	2310	2015–2019	52.9 months	Clear margins Indication to adjuvant radiotherapy including boost	Physician choice (conventional fractionation with SIB or sequential boost or hypofractionated with sequential boost) versus 40 Gy/48 Gy/16 fractions	5‐year local control: 98.2% (experimental) vs. 98.0% (control), *p *= 0.84 Cumulative incidence of Gr 2 ≥ fibrosis at 5 years: 7.3% (experimental) vs. 8.4% (control), *p* = 0.032 Gr 2 telangiectasia at 5 years: ≥ 1.5% (experimental) vs. 1.5% (control)

**Figure 1:**
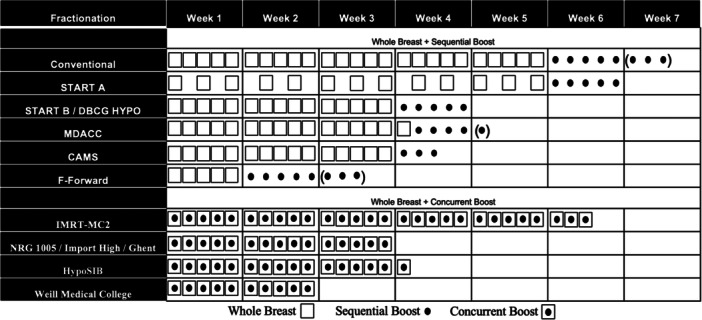
Conventional, moderate, and ultra‐hypofractionated whole breast radiation therapy regimens with or without sequential and concurrent boost used in Phase III prospective trials. Parentheses represent additional variations in boost fractionation schemes on trial.

The first published Phase III trial of an integrated boost was the prospective, multicenter IMRT‐MC2 trial. In this trial, patients were planned to undergo whole breast IMRT using conventional fractionation to 50.4 Gy in 28 fractions and randomized to SIB to 64.4 Gy versus sequential boost to 66.4 Gy in 8 fractions [40]. With a median follow‐up of 5.1 years, the 2‐year local control for the SIB arm was found to be noninferior to the sequential boost arm (99.6% vs. 99.6%, *p* = 0.487) [40]. Cosmetic assessments were performed by physicians and patients using the Harvard criteria. The physician rated cosmetic outcomes were significantly worse after 2 years compared to 6 weeks (*p *= 0.034). In contrast, patients did not classify cosmesis differently between the two time points (*p *= 0.268). The trial was able to demonstrate noninferiority with respect to cosmesis after IMRT‐SIB and 3‐D‐CRT‐seqB at both 6 weeks (median pBRA, 9.1% vs. 9.1%) and 2 years (median pBRA, 10.4% vs. 9.8%) after radiation therapy (*p *= 0.332). The RTOG 1005, which has been presented in abstract form, used moderate hypofractionated WBRT with SIB compared to moderate or conventional WBRT with a sequential boost [41]. With a median follow‐up of 7.4 years, the 7‐year IBTR was noninferior (2.6% vs. 2.2%) [41]. The treatments were well tolerated with no significant differences in Grade 3+ toxicity (*p *= 0.79) [41]. The authors also demonstrated noninferiority for the 3‐year change in baseline of the mean physician‐assessed and patient‐assessed CO between the arms [41]. We await the final publication of RTOG 1005. The IMPORT HIGH trial was a three‐arm randomization between patients treated with 40 Gy in 15 fractions with a sequential boost to 16 Gy in 8 fractions, 40/48 Gy SIB in 15 fractions, or 40/53 Gy in 15 fractions. The authors demonstrated noninferiority with the 48 Gy concurrent boost in the 5‐year IBTR (1.9% control vs. 2.0% 48 Gy boost) [42]. There was no benefit with dose‐escalation to 53 Gy [42]. Figure 1 represents different fractionation and boost regimens used in Phase III prospective trials [26, 34, 36, 40, 41, 42, 43, 44, 45, 46, 47].

The HYPOSIB trial is testing moderate hypofractionation with SIB versus sequential boost [46]. HYPOSIB is a multicenter, noninferiority trial comparing 40 Gy to the breast and 48 Gy to the tumor bed in 16 fractions to the physician's choice of conventional with SIB or sequential boost or hypofractionation with sequential boost. The early results have recently been presented in abstract form. The authors report that the noninferiority of the experimental arm was established with local control at 5 years at 98.2% for the experimental arm and 98.0% in the control arm (*p *= 0.84) [46]. They report cumulative rates of Grade ≥ 2 fibrosis (7.3% vs. 8.4%) and telangiectasia (1.5% vs. 1.5%) at 5 years. We eagerly await the finalized publication.

Ultimately, several large Phase III trials have demonstrated acceptable oncologic and cosmetic results associated with the use of concurrent boost. New data continue to emerge examining the use of concurrent boost in the setting of hypofractionation. SIB provides an opportunity to reduce the duration of treatment. However, the employment of a SIB is not always feasible, typically in cases of a large lumpectomy bed relative to the size of the whole breast at the time of simulation.

## 6 Radiation Technique and Boost

### 6.1 Target Volume Delineation and Patient Set Up

Traditional methods for defining a TBB include delineation of the surgical incision during CT simulation followed by delineation of the postoperative seroma cavity and implanted surgical markers or clips placed intraoperatively on CT scan to define the boost gross tumor volume (GTV). An expansion of 1 cm is used to achieve the clinical target volume (CTV) that is then cropped within the breast as employed on NRG 1005. A planning target volume (PTV) is defined as the CTV plus an additional 5–7 mm margin cropped within the breast to account for setup uncertainty. Patients should be optimally positioned using immobilization devices such as alpha cradle casts, breast boards, and/or wing boards at the time of simulation and treatment to minimize motion and setup uncertainty. Portal films should be performed at the start of treatment if using 3D‐CRT with an orthogonal pair. NRG 1005 suggests subsequent treatments should have at a minimum one set of orthogonal pair images obtained every 5 fractions.

Identification of the seroma cavity is more of a challenge in the setting of oncoplastic breast surgery, where the lumpectomy cavity is closed with tissue re‐approximation to improve cosmetic outcomes. As a result, a seroma does not form. Further, tissue flaps may be rotated to fill the lumpectomy cavity such that the true tumor bed margins could be displaced from the original lumpectomy cavity [48]. Currently, there is no consistent practice for clip placement that has been agreed upon by breast surgeons [48]. There have been several recently published Delphi Consensus statements published by Italian and Canadian experts which have recommended: (1) at a minimum, 4 clips be placed along the 4 side walls (medial, lateral, superior, inferior) of the cavity with 1–4 additional clips at the posterior margin if necessary, (2) minimization of use of clips elsewhere in the breast to improve target delineation, (3) the importance of “speaking a common language” between radiation oncologists and surgeons, and (4) careful consideration as to the value of a boost given the large volume that may need to be included in the setting of oncoplastic breast surgery [48, 49].

Ultimately, size and location of the tumor bed volume may influence the selection of sequential or concurrent TBB. For patients with large seromas, a sequential TBB may be preferred. In RTOG 1005, it was specified that the whole breast receiving the boost dose was required to be under ≤ 30%. The IMPORT HIGH trial stipulated that the tumor bed CTV be ≤ 5% of the whole breast PTV. While large seromas make concurrent boosting challenging, they often reduce in size with time and may even resolve altogether. Hence, the seroma may shrink sufficiently to perform sequential boost by re‐simulation and planning several weeks after starting the WBRT. Further, use of IMRT over 3D‐CRT may mitigate the amount of breast receiving boost dosing. Additionally, if the cavity is superficial and an electron TBB plan is employed, a sequential boost may be more practical. Lastly, some patients may benefit from a boost set up position different from the whole breast component of their whole breast component of their treatment. In these cases, a sequential boost is preferred over a concurrent boost.

The NRG 1005 was the first national breast radiation trial to use 3D planning and target volume contouring, with central quality assurance (QA). In total, 33% of credentialing cases reviewed had deviations in protocol for the lumpectomy surgical cavity boost PTV, and of these, 10% had unacceptable deviations. For subsequent QA reviews of enrolled cases, 29% of the rapid review initial failures were due to an unacceptable deviation to the boost PTV. This suggests that further education and use of standardized atlases are needed to broadly improve the quality of boost radiation oncology treatment planning.

### 6.2 Treatment Modality Selection

Over the past several decades, multiple techniques to deliver boost have been developed in the preoperative, intraoperative (IORT), and postoperative setting. These include intracavitary brachytherapy, various intraoperative radiotherapy (IORT) approaches using electron or low‐energy X‐rays, and various external beam approaches using 3D conformal, intensity modulated radiotherapy, or stereotactic radiosurgery techniques.

**Figure 2:**
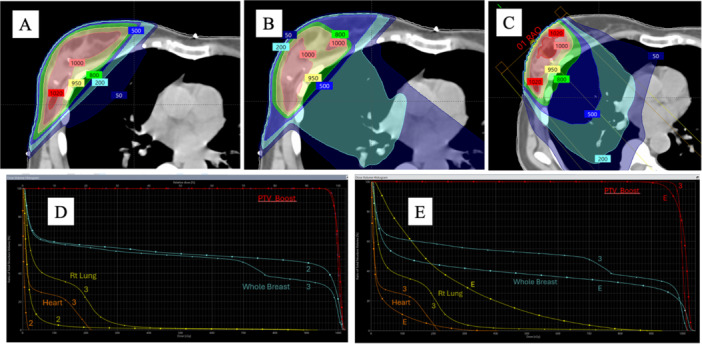
(A) A tangential (2‐field) photon boost covers the lumpectomy bed but has a greater dose to the medial breast outside of the boost target. (B) A three‐field photon boost has a dose plan that is in‐between (A) and (C), having less dose to the medial breast and lower dose to the lung than electrons. (C) An electron boost covers a lumpectomy bed with sparing of breast tissue medially. However, there is a moderate dose exit into the lung posteriorly due to the higher energy of the electron needed for the deeper cavity coverage. (D) Dose volume histogram comparing 2‐field (“2”) and 3‐field boost (“3”). (E) Dose volume histogram comparing 3‐field (“3”) and electron boost (“E”).

The most common boost approach is the employment of an external beam radiotherapy technique. This is often favored due to its noninvasive nature. The technique of radiation boost chosen must weigh the coverage of the target boost planning volume against other factors such as dose homogeneity, dose to the whole breast outside the boost volume, and dose to critical normal organs (e.g., lung or heart) (see Figure 2). A photon boost will, in general, have better dose homogeneity and dose coverage than an electron boost. An electron boost will, by nature of the depth dose characteristics, have greater skin dose than photons, particularly with the higher electron energies, which could have negative cosmetic effects. An electron boost is also limited by the energies adequate for the depth of the lumpectomy bed—usually < 4–5 cm even with the highest electron energies—and has a narrower coverage width at depth compared to photons. A photon boost may give more dose to the whole breast tissue outside the boost volume compared to an electron beam that is *en face* to the breast. A usual limit placed on the boost during planning is 95% of the boost dose should be given to < 25%–30% of the whole breast. This is meant to reduce the incidence of fibrosis, edema, or other boost complications. A photon boost could also potentially give greater exit dose to underlying heart or lung when angled in nontangential directions, but electron beams of higher energies could also exit into the heart if care is not taken during planning as well.

In the modern era, with deep inspiratory breath hold and beam arrangements positioned to avoid the heart, heart doses associated with 3D‐CRT are often extremely low. The NRG 1005 permitted any combination of boost with WBRT (including electrons or photons) except protons or brachytherapy [50]. While in the past electron boosts were most common, in the modern era, the vast majority of cases are photon boosts. In the NRG 1005, approximately 25% of boosts used electrons and 75% used photons. In many treatment centers, electrons may not be available due to equipment capability requiring use of photons in all cases. Further, SIB treatment with electrons requires daily mounting of an electron cone, which can slow clinical workflow, decrease patient throughput, and pose safety risks for radiation therapists.

Brachytherapy represents an alternative boost approach. While brachytherapy may have the best dose fall off, these approaches are invasive and may require higher technical proficiency. Interstitial brachytherapy was included and used by several institutions participating in the EORTC 22881‐10882 trial [31]. Recent studies employing HDR brachytherapy boosts have identified the main risk factor for the development of postradiation fibrosis as the brachytherapy implant volume [51, 52]. An analysis by Wronczewka and colleagues demonstrated that V100, V150, and V200 are most commonly responsible for fibrosis following interstitial HDR brachytherapy [53]. In one study, multicatheter interstitial brachytherapy boost for deeply seated lumpectomy beds limited dose to OARs to a greater degree than three‐field 3D‐CRT and high energy electrons in deeply‐seated lumpectomy beds [54]. However, three‐field 3D‐CRT is not commonly employed in the modern era as it may increase heart and lung doses, so results should be interpreted with caution. One retrospective study investigating whether the boost technique influences the magnitude of local recurrence rates in 1448 tumors found there were no significant differences in local or any recurrence between brachytherapy boost and the electron group or photon group [55].

Utilization of IORT as a boost is an emerging modality with a paucity of literature. IORT approaches have an advantage of being a single‐day treatment and the ability to directly visualize the tumor bed, though not having prior knowledge of margin status prior to treatment remains a challenge. In the setting of oncoplastic tissue rearrangement, IORT may also be beneficial as tumor cavity may be challenging to identify postoperatively. Several studies have reported experiences using intraoperative electrons or use of 50 kV intraoperative boost [56, 57]. The use of low‐energy X‐ray based devices offers better depth penetration compared to their electron counterparts. Unfortunately, exact anatomic dose distributions using these techniques are unknown; therefore, complete dosimetry with subsequent whole breast radiation is not feasible. A randomized trial comparing the use of IORT boost or EBRT boost with postoperative whole breast irradiation (TARGIT‐B trial) is underway. We look forward to seeing the results of this trial.

## 7 Future Directions

There have been new attempts to shorten the length for WBRT and TBB even further. The Weill Cornell Medical Center (WCMC) trial is a Phase III trial comparing M‐WBRT (40 Gy in 15 fractions) with concurrent boost (48 Gy) in 3 weeks versus M‐WBRT (32 Gy in 10 fractions) and concurrent boost (42 Gy) in 2 weeks (NCT04175210). The shortest possible regimen of WBRT and SIB may come from the Chinese Academy of Medical Sciences, which recently reported early results of their 26 Gy in 5 fractions to the whole breast with and without a 6 Gy SIB boost in early‐stage breast cancer. In their initial report, they demonstrated the feasibility of this technique [58]. The prospective MO‐0797 also used WBRT with ultra‐hypofractionation of 26 Gy in 5 fractions with an SIB to 29 Gy. There was no significant increase in acute toxicity at the initial 6‐month report [59].

## 8 Conclusions

Current indications for boost include individuals at elevated risk for IBTR such as those younger than 50 years, high‐grade disease, unfavorable histology (i.e., triple negative), or a focally positive margin. Patient selection remains critical, with increasing data suggesting de‐escalation of boost therapy with modern surgical techniques, and adequate treatment of many patients who would previously be given a boost with instead partial breast radiation. Radiation boost can be delivered sequentially with acceptable toxicity in patients undergoing WBRT. Recent evidence has also emerged supporting the use of concurrent boost in post‐BCS early‐stage breast cancer patients with favorable results. SIB provides an opportunity to further reduce the duration of treatment. We suggest 40 Gy in 15 fractions with SIB to 48 Gy as a convenient schedule. Radiation modality must be selected by weighing individual patient factors to optimize tumor bed coverage while sparing normal breast tissue. In patients with a low risk of IBTR, a TBB can safely be omitted.

## Funding

The authors received no specific funding for this work.

## Synopsis

This focused review summarizes the current status of the post‐breast conservation surgery tumor bed boost for early‐stage breast cancer. We discuss the efficacy of tumor bed boost on ipsilateral breast tumor recurrence, indications for tumor bed boost, cosmetic outcomes of tumor bed boost with standard and hypofractionated radiation, and outcomes using simultaneous integrated boost approach. Lastly, we examine technical considerations for boost planning and delivery.

## Data Availability

Data sharing is not applicable to this article as no data sets were generated or analyzed during the current study.
